# Enzymatic Activity and Amino Acids Production of Predominant Fungi from Traditional Meju during Soybean Fermentation

**DOI:** 10.4014/jmb.2309.09008

**Published:** 2023-12-26

**Authors:** Dong Hyun Kim, Byung Hee Chun, Jae-Jung Lee, Oh Cheol Kim, Jiye Hyun, Dong Min Han, Che Ok Jeon, Sang Hun Lee, Sang-Han Lee, Yong-Ho Choi, Seung-Beom Hong

**Affiliations:** 1 Korean Agricultural Culture Collection, Agricultural Microbiology Division, National Institute of Agricultural Sciences, Wanju 55365, Republic of Korea; 2 Department of Microbiology, Pukyong University, Busan 48513, Republic of Korea; 3 Fermentation Research Lab., Fermentation R&D Center, Sempio Foods Company, Cheongju 28156, Republic of Korea; 4 Department of Life Science, Chung-Ang University, Seoul 06974, Republic of Korea; 5 Food and Nutrition Div., National Institute of Agricultural Sciences, Wanju 55365, Republic of Korea; 6 Department of Food Science and Biotechnology, Kyungpook National University, Daegu 41566, Republic of Korea

**Keywords:** Meju soybean fermentation, fungi, enzyme activity, amino acid

## Abstract

To investigate the effect of the predominant fungal species from Korean traditional meju and doenjang on soybean fermentation, the enzymatic activity and amino acid production of twenty-two fungal strains were assessed through solid- and liquid-state soybean fermentation. Enzymatic activity analyses of solid-state fermented soybeans revealed different enzyme activities involving protease, leucine aminopeptidase (LAP), carboxypeptidase (CaP), glutaminase, γ-glutamyl transferase (GGT), and amylase, depending on the fungal species. These enzymatic activities significantly affected the amino acid profile throughout liquid-state fermentation. Strains belonging to *Mucoromycota*, including *Lichtheimia*, *Mucor*, *Rhizomucor*, and *Rhizopus*, produced smaller amounts of total amino acids and umami-producing amino acids, such as glutamic acid and aspartic acid, than strains belonging to *Aspergillus* subgenus *circumdati*. The genera *Penicillium* and *Scopulariopsis* produced large amounts of total amino acids and glutamic acid, suggesting that these genera play an essential role in producing umami and kokumi tastes in fermented soybean products. Strains belonging to *Aspergillus* subgenus *circumdati*, including *A. oryzae*, showed the highest amino acid content, including glutamic acid, suggesting the potential benefits of *A. oryzae* as a starter for soybean fermentation. This study showed the potential of traditional meju strains as starters for soybean fermentation. However, further analysis of processes such as the production of G-peptide for kokumi taste and volatile compounds for flavor and safety is needed.

## Introduction

Fermented soybean foods, including doenjang, ganjang, doubanjiang, douchi, hawaijar, cinema, miso, natto, and soy sauce, are popular in many Asian countries [1-4]. These foods are prepared by fermenting soybeans with various microorganisms, including bacteria and fungi, to improve their nutritional value and flavor [5-8].

During the production of various types of jang, a type of Korean traditional fermented food ingredient that includes among others ganjang (soy sauce) and doenjang (soybean paste), fungi and bacteria contribute to the taste- and flavor-related metabolites, thereby enhancing the quality of the products [9-12]. Recently, diverse bacterial strains identified in meju (block of traditional Korean fermented soybean) have been reported to influence the levels of volatile flavoring compounds during fermentation [13]. However, studies on the fermentation characteristics of the various fungi found during soybean fermentation have yet to be conducted extensively.

Fungi play an essential role in the breakdown of high-molecular-weight metabolites, including proteins, carbohydrates, and lipids, during soybean fermentation [9-12]. In industrial processes, restricted fungal strains of *Aspergillus oryzae* or *A. sojae* are used as starters to hydrolyze proteins, lipids, and starch. However, in traditional fermentation processes, there are no specific fungal strains for inoculation, allowing a variety of fungi to be inoculated during fermentation. A few studies have reported the predominant fungal species involved in meju fermentation using culturomic and metagenomic analyses, and *Aspergillus*, *Penicillium*, *Scopulariopsis*, and *Mucoromycota*, including *Lichtheimia*, *Mucor*, *Rhizomucor*, and *Rhizopus*, were identified as predominant fungi in meju [14-16, 38]. These diverse fungal species originating from meju contribute to the unique taste and aroma of jang, which uses meju as an ingredient [17, 18]. Previous research has primarily focused on *Aspergillus* metabolism during industrial fermentation. Recent studies have investigated the fermentation characteristics of soybeans using fungal strains belonging to the genera *Penicillium*, *Rhizopus*, and *Mucor* [17, 19]. However, research on the predominant fungal species observed during the natural fermentation of soybeans, such as *Lichtheimia*, *Cladosporium*, *Monascus*, *Scopulariopsis*, and *Rhizomucor*, remains limited, and further studies on these diverse fungi are therefore required [15, 16, 20].

Soybean fermentation can be divided into solid- and liquid-state fermentations [5]. In Korea, solid-state fermentation, or meju fermentation, is a technique in which microbial fermentation occurs without adding free water during manufacturing [21]. During solid-state fermentation, fungi exhibit high enzyme activity and produce enzymes that contribute to the decomposition of high-molecular-weight materials [22-24]. Liquid-state fermentation, also known as the ripening stage, is performed by adding the fermented soybeans produced from the solid-state fermentation process to brine, followed by additional fermentation [25]. Fungal growth is reduced in liquid-state fermentation, and enzymes including protease, peptidase, and amylase, which originated from the fungi during solid-state fermentation, play a major role in the degradation of high-molecular-weight materials and generate metabolites related to taste and flavor, such as free amino acids and aglycons [26]. Therefore, investigating the fungal activity in fermented soybeans is essential for understanding the degradation and production of major metabolites in liquid- and solid-state fermentation of soybeans.

In this study, 20 strains of fungi predominantly identified from traditional meju and two fungal strains used in industry were used as starters for solid-state and liquid-state fermentation of soybeans, and their enzymatic activity and amino acid production of fermented soybeans were analyzed to elucidate the effects of the predominant meju fungi on soybean fermentation.

## Materials and Methods

### Preparation of Twenty-two Fermented Soybean Samples Made with Diverse Fungal Starters

Twenty-two fungal strains were used for soybean fermentation. Twenty fungal strains were isolated from Korean fermented soybeans (meju), and two fungal strains were obtained from a processed fermented soybean product called doenjang. Briefly, twenty strains from genera *Lichtheimia*, *Mucor*, *Rhizomucor*, *Rhizopus*, *Cladosporium*, *Penicillium*, *Scopulariopsis*, *Monascus*, *Aspergillus* subgenus *Aspergillus* (*A. pseudoglaucus*, *A. tonophilum*, *A. chevalieri*) and *Aspergillus* subgenus *Circumdati* (*A. candidus*, *A. oryzae*, *A. luchuensis*) isolated from traditional meju or doenjang samples were collected from the KACC (Korean Agricultural Culture Collection), and two strains (from genus *Aspergillus*) were collected from Chungmu Fermentation Ltd. (Republic of Korea) (Table 1) [16, 27]. All fungal strains were cultured on malt extract agar media (MEA, Oxoid Ltd., UK) at optimal temperatures and inoculated as starters for soybean fermentation.

Solid-state fermentation of raw soybeans with 22 different fungal starters was performed according to the manufacturer’s protocol (Sempio Foods Company, Republic of Korea), with certain modifications (Fig. S1). Briefly, a total of 1.2 kg of soybean was moistened (50%, w/v), sterilized (121°C, 15 min), and divided into 66 flasks. After cooling the soybean, twenty-two kinds of fungal starters were inoculated into each flask in triplicate as 6.5 mm discs of mycelia and incubated at the optimal temperature of the inoculated fungal strain for 10 days. The fermented soybean samples were collected from each flask at different time points (days 2, 4, 6, 8, and 10), crushed using a homogenizer, and stored at −80°C for further analyses. To assess the fungal enzyme activity, including protease, leucine aminopeptidase (LAP), carboxypeptidase (CaP), glutaminase, γ-glutamyl transferase (GGT), and amylase in fermented soybean samples, an enzyme in fermented soybean samples was extracted according to a previously described procedure with some modifications [28-30]. Briefly, 10 g of fermented soybean sample was dissolved in 190 ml of a 2% NaCl solution and stirred (at 200 rpm) for 15 min at 25°C. The supernatant was filtered using filter paper No. 2 (Whatman, UK) and stored at −80°C for further analyses.

### Measurement of Fungal Biomass during Soybean Fermentation

The quantity of fungal biomass was estimated by calculating the chitosan and chitin contents in the fermented soybean samples according to a previously described procedure, with certain modifications [31,32]. Briefly, 10 mg of fermented soybean samples was suspended in 60% sulfuric acid and reacted at 121°C for 1 h. Then, 0.5 ml of 1M NaNO_2_ and 0.5 ml of ammonium sulfamate solution (12%, w/v) were added to the fermented soybean solution, and the mixture was reacted at room temperature for 6 h. After adding 0.5 ml of 0.5% 3-methyl-2-benzothiazolinone hydrazone hydrochloride (MBTH) and 0.5 mL of 0.5% FeCl_3_ into the mixture, the quantities of chitosan and chitin were estimated by measuring the absorbance at 650 nm and quantified using glucosamine as a standard.

### Measurement of Enzymatic Activities for Protein Metabolism in Fermented Soybean Samples

To analyze the enzymatic activity for the hydrolysis of neutral protein, 1 ml of enzyme extract from a fermented soybean sample was added to 2 ml of 100 mM phosphate buffer containing casein (2%, w/v) and incubated at 30°C for 20 min. After the reaction, 5 ml of 0.4 M trichloroacetic acid solution was added to the enzyme extract solution for 20 min at room temperature and filtered through filter paper No. 4 (Whatman). Finally, 1 ml of filtrate, 5 ml of 0.4 M Na_2_CO_3_, and 1 ml of Folin’s reagent (Sigma-Aldrich, USA) were mixed and incubated for 20 min at 30°C. The enzymatic activity for the hydrolysis of neutral proteins was estimated by measuring the differences in absorbance at 660 nm and quantified using L-tyrosine as a standard. One Unit (U) of protease activity was defined as the amount of enzyme that produces 1 μg of tyrosine in 1 min.

Leucine aminopeptidase activity was determined by measuring the amount of p-nitroaniline released from L-leucine-p-nitroanilide using spectrophotometry. Briefly, 0.1 ml of enzyme extract from a fermented soybean sample was mixed with 2.8 ml of 50 mM sodium phosphate buffer (pH 7.0) and 24 mM L-leucine-p-nitroanilide (0.1 ml) and incubated at 50°C for 10 min. The absorbance was measured at 405 nm to investigate the production of p-nitroaniline and was compared with a standard curve of p-nitroaniline. One U of LAP activity was defined as the amount of enzyme that released 1 μmol of p-nitroaniline in 1 min.

To analyze the enzymatic activity of CaP, 0.1 ml of enzyme extract from a fermented soybean sample was mixed with 0.1 ml of 0.5 M acetate buffer (pH 3.0) and 1 ml of 0.5 mM Cbz-Glu-Tyr and incubated at 30°C for 20 min. After 0.5 ml of ninhydrin was added, the mixture was boiled for 15 min, cooled quickly, and 5 ml of 60% ethanol was added. The enzymatic activity of CaP was estimated by measuring the absorbance of the mixture at 570 nm and quantified using L-tyrosine as a standard. One U of protease activity was defined as the amount of enzyme that produces 1 μmol of tyrosine in 1 min.

### Measurement of Enzymatic Activities for Taste and Functionality of Fermented Soybean during Fermentation

Glutaminase activity was determined by measuring glutamate production from glutamine. Briefly, 0.2 ml of enzyme extract from a fermented soybean sample was mixed with 0.7 ml of 100 mM Tris-HCl buffer (pH 7.5) and 0.2 ml of 100 mM L-glutamine and incubated at 45°C for 30 min. Glutamate production in the mixture was measured using a Cedex Bio-Bioprocess analyzer (Roche Ltd., Switzerland) and quantified using L-glutamic acid as a standard. The activity of glutaminase was determined as 1 U by measuring the rate of L-glutamic acid production from L-glutamine per minute, resulting in the release of 1 μmol.

γ-Glutamyle transferase activity was determined by measuring the production amount of p-nitroaniline from γ-glutamyl-p-nitroanilide. Briefly, 0.1 ml of enzyme extract from a fermented soybean sample was mixed with 0.9 ml of 50 mM sodium phosphate buffer (pH 8.0) containing 1 mg of γ-glutamyl-p-nitroanilide and incubated at 37°C for 60 min. The amount of p-nitroaniline produced in the mixture was estimated by measuring the absorbance at 418 nm and quantified using p-nitroaniline as a standard.

To analyze the amylase activity, 1 ml of enzyme extract from a fermented soybean sample was mixed with 4 ml of citrate buffer (pH 5.2) containing starch (0.5%, w/v) and incubated at 40°C for 30 min. After adding the iodine solution, amylase activity was estimated by measuring the absorbance at 660 nm and quantified using starch as a standard material. The amylase activity (U) was quantified by assessing the rate of starch degradation at a rate of 1mg/min.

### Preparation of Fermented Soybean Solution Made by Liquid-State Fermentation of Fermented Soybean Samples for Amino Acid Content Analysis

The fermented soybean samples were further ripened in a solar salt solution for liquid fermentation according to a previously described procedure with certain modifications. Briefly, 30 g of fermented soybean sample collected at different fermentation time points (2 and 4 days) was mixed with 70 ml of solar salt water (9.6% w/v) and incubated at 41.5°C for 2 days. After 2 days of fermentation, the liquid from the fermented soybean solution was collected and stored for further analysis.

The amino acid content in the fermented soybean solution prepared from fermented soybeans on days 2 and 4 of fermentation was analyzed according to a previously described method with certain modifications (MFDS 2022). Briefly, 1 ml of fermented soybean solution was mixed with 10 ml of 6 N HCL, reacted at 105°C for 1 day, and washed with sample dilution buffer (Sykam, 0.12 N; pH 2.20). The amino acids were analyzed using an analyzer (S 433, Sykam).

## Results

### Characteristics of Fungal Starters during Soybean Fermentation

To investigate the effect of the predominant fungal species from traditional meju on soybean fermentation, 18 fungal strains isolated from meju and two strains from doenjang were obtained from the KACC. Two commercial fungal strains (CF1005 and CF1001) were obtained from an industrial fermentation company, and their optimal growth temperatures were investigated (Table 1). In total, twenty species in Table 1, including nine genera, were collected, and the optimal growth temperature of the twenty-two fungal strains ranged from 25 to 40°C. Individual fungal strains were inoculated into soybeans and incubated at the optimal growth temperature of 10 days for soybean fermentation (Fig. S1). The fungal biomass in the fermented soybean samples was estimated by calculating the amounts of chitosan and chitin in the samples [31]. In most of the fermented soybean batches, the fungal biomass increased as fermentation progressed and showed similar amounts among the same genera, except for *R. delemar* SP7 in Table 1. The fungal biomass of *R. oryzae* after 6 days of soybean fermentation was the highest (approximately 29.9 μg/mg soybean) among all fermented soybean samples. Despite differences in fermentation temperatures and the composition of the starter species, variations in the quantity of fungal biomass across the eight batches produced by strains belonging to the genus *Aspergillus* (SP15-22) exhibited only minimal similarities during soybean fermentation (Table S3).

### Enzyme Activities for Protein Metabolism during Soybean Fermentation

**Fig. 2:**
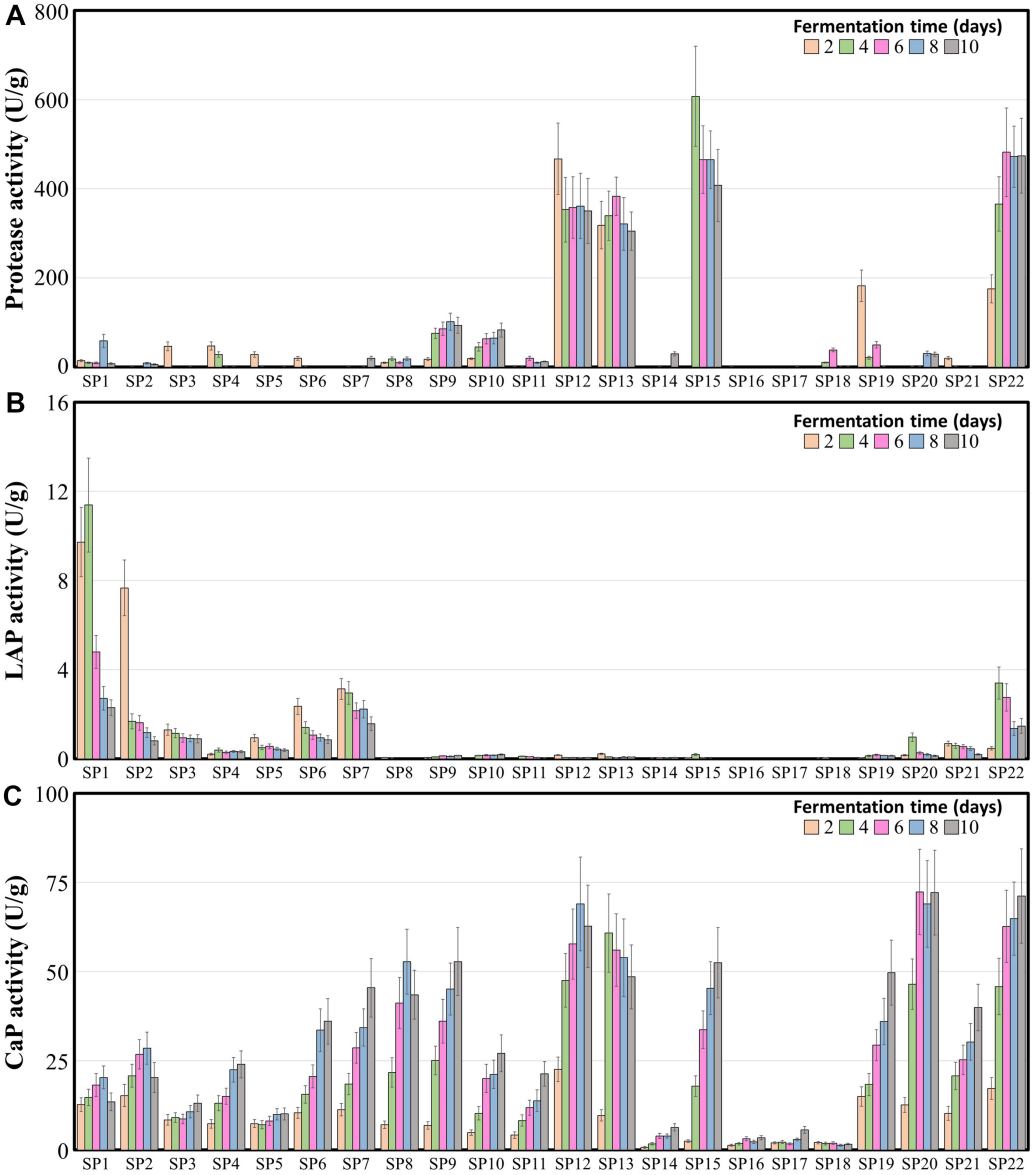
Profiles of protease (A) leucine aminopeptidase (B) and carboxypeptidase (C) activities in fermented soybean during solid-state fermentation process. For each strain, five samples are gathered at different time points (2, 4, 6, 8 and 10 days). The enzyme activity was determined by measuring the change in optical density (OD) as each enzyme degraded its corresponding substrate. The error bars indicate standard errors; data were measured in triplicate.

As protein is a major high-molecular-weight metabolite in soybeans, measuring the protein-degrading ability of the fungal starters is important for evaluating their fermentation characteristics [9, 33]. The protease activity of the twenty-two fungal strains was assessed by measuring the hydrolytic activity of casein (Fig. 2A). Assessment of protease activity showed similar results for fermented soybean samples inoculated with fungal starters belonging to the same genera, except for the genus *Aspergillus*. The protease activity in fermented soybean samples produced by fungal strains, including the genera *Scopulariopsis* strains (SP12 and SP13) and *Aspergillus* subgenus *Circumdati* strains (SP15 and SP22), was high (over 305.0 U at 10 days of fermentation), suggesting that these fungal strains can serve as key starters because of their strong protein-degrading capabilities. The protease activity in samples produced by fungal strains belonging to the genus *Penicillium* (SP9 and SP10) was lower (approximately 90.0 U at 10 days of fermentation), and the protease activity in fermented soybean produced by the other strains was significantly low (less than 60.0 U at 10 days of fermentation) during the entire fermentation period (Table S4). Exopeptidase activity at the N- and C-termini in the fermented soybean samples was assessed by measuring LAP and CaP activity, respectively (Fig. 2B and 2C). Exopeptidase activity assessment of LAP and CaP showed that LAP was highly expressed during the early soybean fermentation period. In contrast, CaP was highly expressed during the middle and late soybean fermentation periods. The LAP activity in samples produced by the genera *Lichtheimia* (SP1 and SP2) was significantly higher (over 7.7 U at 4 days of fermentation) than in samples produced by other fungal strains during the early soybean fermentation period and decreased as fermentation progressed (Table S4). The LAP activity in samples produced by strains in *Mucoromycota*, including *Mucor* (SP3 and SP4), *Rhizomucor* (SP5), and *Rhizopus* (SP6 and SP7), and by certain strains in the genus *Aspergillus* subgenus *Circumdati* (SP20, SP21, and SP22), was rather low, while LAP activity in fermented soybeans produced by other fungal strains was significantly low during the entire soybean fermentation period. The CaP activity in most of the fermented soybean samples was high, except for the fermented soybean produced by a strain belonging to the genus *Monascus* (SP14) and a few strains belonging to *Aspergillus* subgenus *Aspergillus* (SP16, SP17, and SP18), which was previously named genus Eurotium [34].

### Assessment of Enzymatic Activity Related to the Taste and Functionality of Fermented Soybeans during Fermentation

**Fig. 3:**
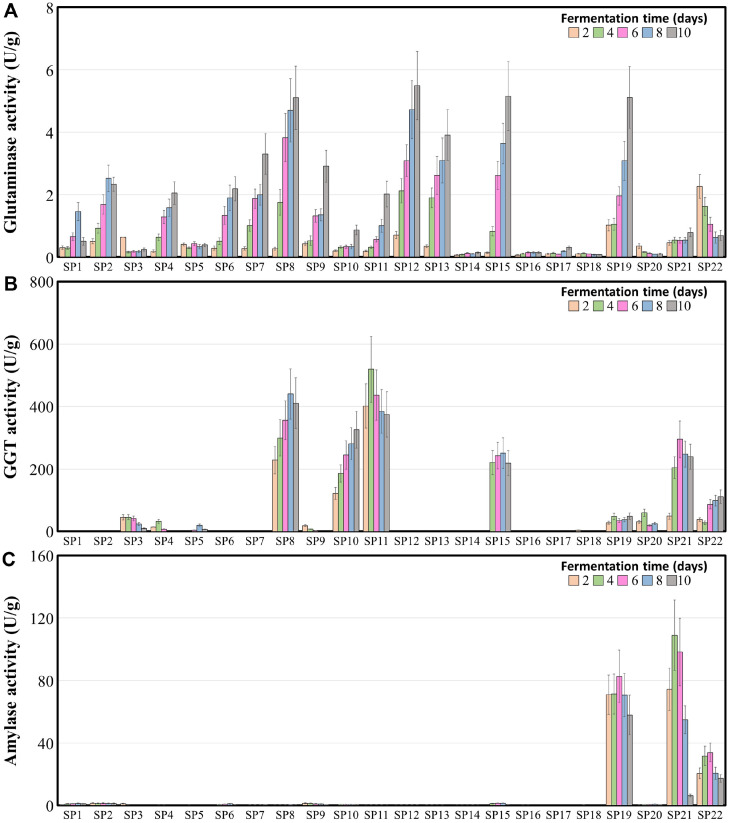
Profiles of glutaminase (A) gamma-glutamyltransferase (B) and amylase (C) activities in fermented soybean during solid-state fermentation process. For each strain, five samples are gathered at different time points (2, 4, 6, 8 and 10 days). The enzyme activity was determined by measuring the change in optical density (OD) as each enzyme degraded its corresponding substrate. The error bars indicate standard errors; data were measured in triplicate.

The representative tastes of traditional Korean soybean-fermented foods, such as umami and kokumi, are mainly related to the production of metabolites, including glutamic acid and γ-glutamyl peptides, respectively, during fermentation [23, 24]. The metabolites, including aglycons and isoflavone, which have been linked to enhancing the functionality of fermented soybean foods, are produced by the enzyme amylase [35]. These enzymes are mainly produced by the fungi present in fermented soybean foods [36]. The enzyme activities of glutaminase, GGT, and amylase responsible for producing glutamic acid, γ-glutamyl peptides, aglycons, and isoflavone, respectively, in fermented soybean samples were assessed (Fig. 3). The enzymatic activity of glutaminase increased as the fermentation progressed (Fig. 3A). The glutaminase activity produced by strains belonging to genus *Cladosporium* (SP8), *Scopulariopsis* (SP12 and SP13), and a few strains belonging to *Aspergillus* subgenus *Circumdati* (SP15 and SP19) was high (above 3.9 U/g at 10 days of fermentation). The glutaminase activity by strains belonging to the genus *Monascus* (SP14), genera *Aspergillus* subgenus *Aspergillus* (SP16, SP17, and SP18), and *Aspergillus luchuensis* SP20 (*Aspergillus* subgenus *Circumdati*) was significantly lower than in the other fermented soybean samples (Table S4).

The assessment of GGT enzyme activity in fermented soybean samples showed that the GGT enzyme activity in fermented soybeans produced by strains belonging to the genera *Cladosporium* (SP8) and *Penicillium* (SP10 and SP11) and certain strains belonging to *Aspergillus* subgenus *Circumdati* (SP15 and SP21) was significantly higher (above 200 U), whereas the GGT enzyme activity in fermented soybeans produced by other fungal strains was much lower during the entire soybean fermentation period (Fig. 3B). The amylase activity was high in fermented soybeans produced by certain strains belonging to *Aspergillus* subgenus *Circumdati* (SP19, SP21, and SP22), whereas it was lower in fermented soybeans produced by other strains (Fig. 3C). The amylase activity in fermented soybeans produced by *A. oryzae* SP19 and *A. luchuensis* SP21 was significantly high (109.0 U at 4 days) (Table S4).

### Amino Acid Production in a Fermented Soybean Solution Made with Twenty-two Fermented Soybean Samples

**Fig. 4:**
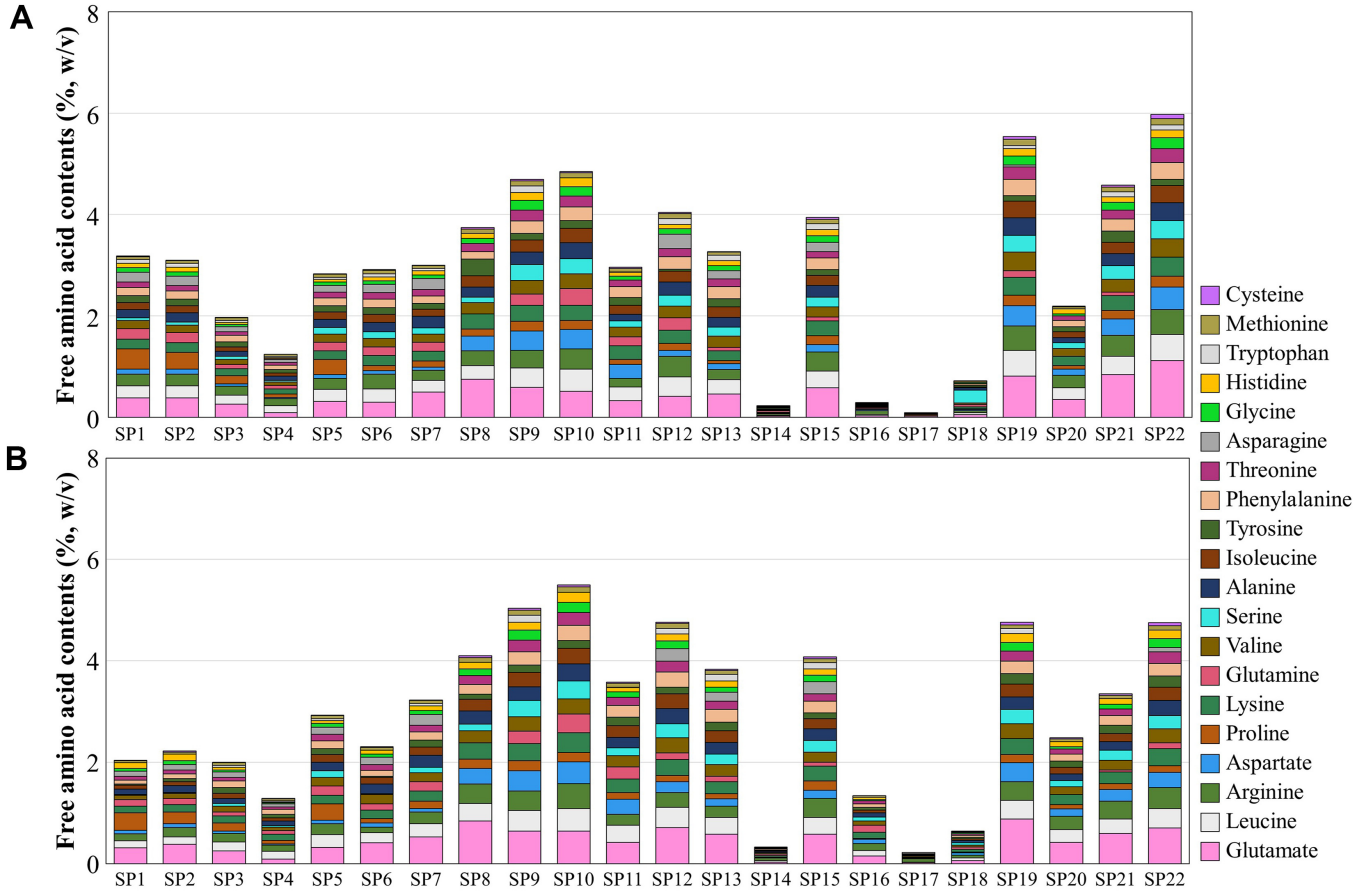
Amino acids contents from fermented soybean solution prepared from fermented soybean on the 2 (A) and 4 (B) days of liquid-state fermentation. The amino acids contents were analyzed using an amino acid analyzer.

To investigate the effect of the predominant fungal species from traditional meju on liquid-state soybean fermentation, the fermented soybean samples at 2 and 4 days were soaked in salt water and fermented for 3 days, and the amino acid content in the fermented soybean solution was analyzed (Table S1). The amino acid profile of fermented soybean solution showed a similar pattern when the fungal starters of the same genera were used, except when the fungal starters belonged to the genera *Aspergillus*. The total amino acid content prepared by strains belonging to the genera *Monascus* (SP14) and *Aspergillus* subgenus *Aspergillus* (SP16, SP17, and SP18), which have low protease, LAP, and CaP activity, was significantly low in all fermented soybean samples (Table S5). Glutamic acid is known to have the strongest umami taste among amino acids, followed by aspartic acid. The concentration of glutamic acid in the soybean solution fermented using the fungal strain SP22 after 2 days of solid-state fermentation with high glutaminase activity was the highest among all fermented soybean solution samples (1.1%, w/v) (Fig. 4A). The amount of glutamic acid in fermented soybean solution prepared by strains belonging to genus *Cladosporium* (SP8) and *Aspergillus* (SP19) was high, followed by *Penicillium* (SP 9, 10) and *Aspergillus* subgenus *Circumdati* (SP19, SP21), while the amount of glutamic acid in fermented soybean solution prepared by strains belonging to genus *Mucor* (SP4), *Monascus* (SP14), and *Aspergillus* subgenus *Aspergillus* (SP16, SP17, and SP18) was significantly low in all fermented soybean samples (Table S5). The aspartic acid content was high in *Penicillium polonicum* (SP 9 and 10) and *Aspergillus oryzae* (SP 19 and 22). The amount of proline was high in fermented soybean solutions prepared using strains belonging to the genera *Lichtheimia* (SP1 and SP2) and *Rhizomucor* (SP5), and the amounts of lysine and arginine were high in fermented soybean solutions prepared using strains belonging to the genus *Penicillium* (SP8, SP9, and SP10) and a few strains belonging to *Aspergillus* subgenus *Circumdati* (SP15, SP19, SP20, SP21, and SP22). These results indicated that the fungal starter taxon affected the production of amino acids in fermented soybean solutions.

## Discussion

In the industrial soybean fermentation process, fungal strains of *A. oryzae* are mainly used as starters for soybean fermentation [37]. However, during the natural fermentation process of soybeans, diverse fungi participate in fermentation, suggesting that fungi other than *A. oryzae* can play important roles [24].

Analyses of the enzymatic activity of twenty-two fungal strains during solid-state soybean fermentation revealed that different enzymatic activities, including those of protease, LAP, CaP, glutaminase, GGT, and amylase, are exerted by the fungal species. These enzymatic activities significantly affect the amino acid profile throughout the liquid-state fermentation process and impact the overall quality, indicating that specific fungal strains play a pivotal role in producing high-quality fermented soybean products. The genus *Mucor* usually grows between the cracks of traditional meju during the early fermentation period, and the genus *Rhizopus* has been reported as a significant fungus during the early fermentation period of meju [16]. However, *Lichtheimia* and *Rhizomucor* grow at high temperatures during the late stages of meju fermentation [16]. During meju fermentation, *Mucoromycota* strains exhibit distinctive characteristics, such as robust growth on the meju substrate, making their mycelia highly visible. However, their potential to produce amino acids and taste-associated enzymes through soybean fermentation has yet to be reported. In this study, strains belonging to *Mucoromycota*, including *Lichtheimia*, *Mucor*, *Rhizomucor*, and *Rhizopus*, produced lower amounts of amino acids than strains belonging to *Ascomycota*, except for *Monascus* and *Aspergillus* subgenus *Aspergillus* (Fig. 4). They produced lower amounts of glutamic acid and aspartic acid, which are related to umami, than other fungal strains. However, enzymatic activity analyses showed that the two strains belonging to the genus *Lichtheimia* produced the highest amounts of LAP, suggesting that these strains may contribute to the taste of fermented soybeans. The genus *Penicillium* is commonly found in traditional meju fermentations, as meju fermentation occurs outdoors during the winter, and *Penicillium* grows well at low temperatures [38]. In this study, strain SP10 (*P. solitum*) produced large amounts of total amino acids (5.50%, w/v) and glutamic acid (0.64%, w/v) (Fig. 4A and Table S1). In contrast, strain SP11 (*P. roqueforti*) produced significantly higher levels of GGT (Fig. 3). These results suggest that the genus *Penicillium* could play an essential role in the production of umami and kokumi tastes from fermented soybean products.

The genus *Scopulariopsis* has been identified predominantly in the late meju fermentation period, and the high abundance of genus *Scopulariopsis* on the surface of meju suggests a satisfactory degree of meju fermentation [38]. The strains belonging to the genus *Scopulariopsis* (SP12 and SP13) showed high protease and carboxypeptidase activities during solid-state soybean fermentation (Fig. 2). SP12 (*S. brevicaulis*) produced increased amounts of amino acids (4.77% w/v) and glutamic acid (0.71% w/v) during liquid-state soybean fermentation (Fig. 4B and Table S1). These results suggest that the genus *Scopulariopsis* may play an important role in the taste and flavor of fermented soybeans. *Monascus ruber*, often found in traditional meju during the late stages of fermentation, has been reported to produce monacolin K, which inhibits cholesterol synthesis [38]. In this study, strain SP14 showed significantly lower enzymatic activity and amino acid production than other fungal strains. This suggests that the impact of *M. ruber* on the taste of soybean products was insignificant. In contrast, it may influence the functionality of fermented soybeans through the production of monacolin K.

In this study, *Aspergillus* showed different fermentation characteristics depending on the subgenus. The subgenus *Aspergillus* has been reported to grow under conditions of low water activity, which supports its robust growth on the surface of meju during late fermentation periods (Hong *et al*., 2015). Strains belonging to the *Aspergillus* subgenus *Aspergillus* (SP16, SP17, and SP18) showed significantly lower enzyme activity and amino acid production during soybean fermentation, suggesting that their effects on the taste and flavor of soybean products are incompetent. However, the strains belonging to *Aspergillus* subgenus *Circumdati* (SP15, SP19, SP20, SP21, and SP22) exhibited high enzyme activity and amino acid production during soybean fermentation. Strain SP15 (*A. candidus*) showed significantly higher protease activity during solid-state soybean fermentation and amino acid production during liquid-state soybean fermentation. The SP21 (*A. luchuensis*) industrial strain used for producing makgeolli, a traditional Korean fermented rice wine, showed significantly high amylase activity. In contrast, the SP22 (*A. oryzae*) strain used for producing doenjang and ganjang, a traditional Korean fermented soybean food, showed significantly high protease activity and glutamic acid production. Strains belonging to *A. oryzae* (SP19 and SP22) showed high amino acid contents, including glutamic acid, which enhances the umami taste and suggests the potential benefits of *A. oryzae* as a starter for soybean fermentation,.

In this study, we analyzed the fermenting enzymes after solid fermentation and amino acids after liquid fermentation. Glutamic acid and aspartic acid are crucial components of the umami taste during soybean fermentation. Therefore, the effects of the predominant fungi from traditional meju on umami taste during soybean fermentation could be analyzed. Our findings provide insights into the metabolic characteristics of fungal starter strains during soybean fermentation and potential fungal starter strains that can be used for soybean fermentation. However, further analysis of processes such as the production of G-peptides for kokumi taste and volatile compounds for flavor and safety is needed to determine the quality and safety of soybeans fermented using fungal starters.

## Supplemental Materials

Supplementary data for this paper are available on-line only at http://jmb.or.kr.



## Figures and Tables

**Fig. 1:**
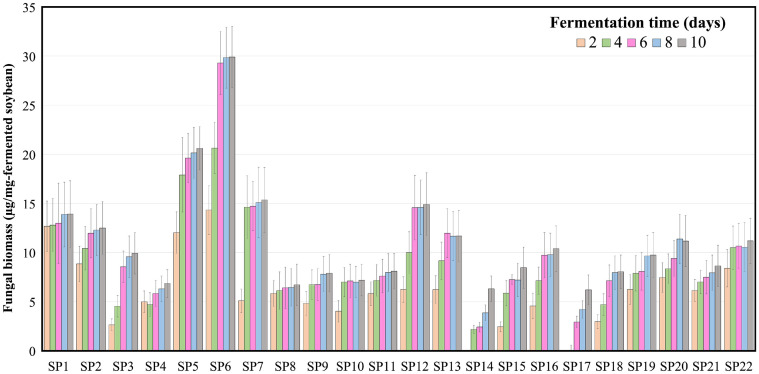
Contents of fungal biomass in twenty-two types of fermented soybean during the fermentation. For each strain, five samples are gathered at different time points (2, 4, 6, 8 and 10 days). The error bars indicate standard errors; data were measured in triplicate.

**Table 1:** List of twenty-two fungal strains used in this study for soybean fermentation and their optimal growth temperature.

No.	Strain name	KACC No.	Isolation source	Isolation region	Op.Temp. (°C)^a^
SP1	*Lichtheimia ramose*	KACC 93209	Meju	Anseong-si Gyeonggi-do	40
SP2	*Lichtheimia ornate*	KACC 45837	Meju	Gyeongsan-si, Gyeongsangbuk-do	40
SP3	*Mucor circinelloides*	KACC 93204	Meju	Icheon-si, Gyeonggi-do	30
SP4	*Mucor mucedo*	KACC 46082	Meju	Yongin-si, Gyeonggi-do	25
SP5	*Rhizomucor pusillus*	KACC 93207	Meju	Yongin-si, Gyeonggi-do	40
SP6	*Rhizopus oryzae*	KACC 46101	Meju	Icheon-si, Gyeonggi-do	35
SP7	*Rhizopus delemar*	KACC 46100	Meju	Gongju-si, Chungcheongnam-do	35
SP8	*Cladosporium cladosporioides*	KACC 93208	Meju	ND	20
SP9	*Penicillium polonicum*	KACC 93205	Meju	ND	25
SP10	*Penicillium solitum*	KACC 93212	Meju	Yangpyeong-gun, Gyeonggi-do	20
SP11	*Penicillium roqueforti*	KACC 48314	Doenjang	Wanju-gun, Jeollabuk-do	25
SP12	*Scopulariopsis brevicaulis*	KACC 93202	Doenjang	Wanju-gun, Jeollabuk-do	30
SP13	*Scopulariopsis candida*	KACC 46678	Meju	Gongju-si, Chungcheongnam-do	30
SP14	*Monascus ruber*	KACC 46645	Meju	Icheon-si, Gyeonggi-do	35
SP15	*Aspergillus candidus* (subg. *circumdati*)	KACC 93203	Meju	Chilgok-gun, Gyeongsangbuk-do	25
SP16	*Aspergillus pseudoglaucus* (subg. *Aspergillus*)	KACC 93211	Meju	ND	30
SP17	*Aspergillus tonophilum* (subg. *Aspergillus*)	KACC 45365	Meju	ND	25
SP18	*Aspergillus chevalieri* (subg. *Aspergillus*)	KACC 46342	Meju	ND	30
SP19	*Aspergillus oryzae* (subg. *circumdati*)	KACC 93210	Meju	Sunchang-gun, Jeollabuk-do	30
SP20	*Aspergillus luchuensis* (subg. *circumdati*)	KACC 46490	Meju	Yeoju-si, Gyeonggi-do	30
SP21	*Aspergillus luchuensis* (subg. *circumdati*)	CF1005	ND	Chungmoo Fermentation Co.	35
SP22	*Aspergillus oryzae* (subg. *circumdati*)	CF1001	Meju	Chungmoo Fermentation Co.	30

^a^The optimal growth temperature was investigated in this study
