# Supplementary material for: Enzymatic Activity and Amino Acids Production of Predominant Fungi from Traditional Meju during Soybean Fermentation
Source: J Microbiol Biotechnol. 2023 Dec 26;34(3):654–62. doi: 10.4014/jmb.2309.09008 (PMC11016766; doi:10.4014/jmb.2309.09008)
Supplement: Supplementary file 1 [file jmb-34-3-654-supple.pdf]

J. Microbiol. Biotechnol.
https://doi.org/10.4014/jmb.2309.09008

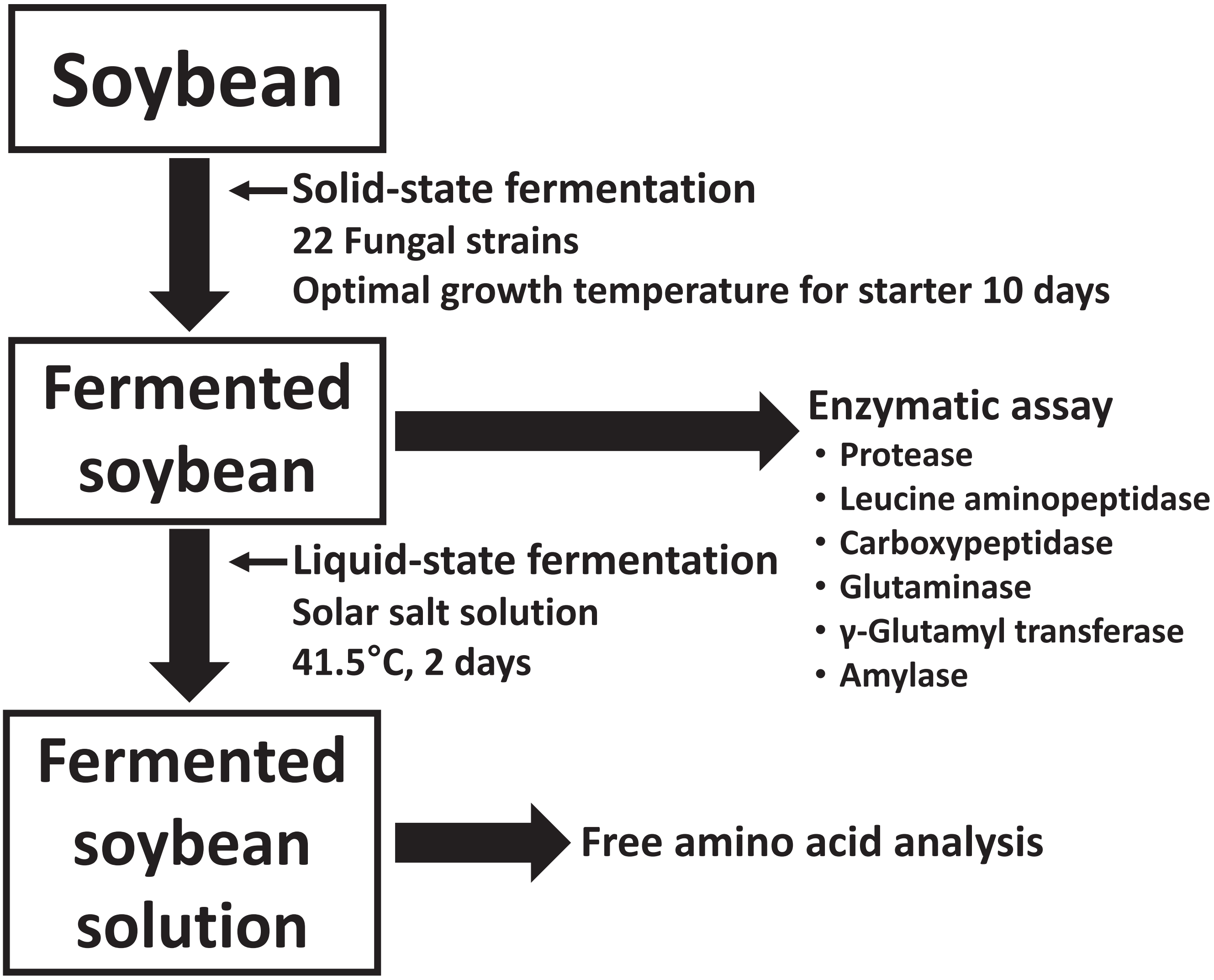

Supplementary Figure 1. Fermentation process of soybean. Enzymatic assay was conducted with 2, 4, 6, 8 and 10 days solid-state fermentation result. Fermented soybean sample collected at different fermentation time points (2 and 4 days) were utilized for liquid-state fermentation and free amino acid analysis is conducted.
